# Life expectancy by ethnic origin in Chile

**DOI:** 10.3389/fpubh.2023.1147542

**Published:** 2023-06-15

**Authors:** Moisés H. Sandoval, Marcela E. Alvear Portaccio, Cecilia Albala

**Affiliations:** ^1^Unidad de Nutrición Pública, Instituto de Nutrición y Tecnología de los Alimentos, Universidad de Chile, Santiago, Chile; ^2^Universidad Nacional de Colombia, Bogotá, Colombia

**Keywords:** life expectancy (LE), ethnic differences, Indigenous, non-Indigenous, Chile

## Abstract

**Background:**

Ethnic and racial differences in life expectancy have been well established in different societies. However, even though an important part of the population of Latin America is Indigenous, there is little knowledge about them.

**Objective:**

Determine if there are ethnic differences in life expectancy at birth and at 60 years in Chile, and if the Mapuche (largest Indigenous ethnic group) have similar life expectancy to other Indigenous peoples.

**Method:**

Life tables for the Mapuche and other Indigenous groups and non-Indigenous people were built using the 2017 census. Specifically, we used the questions of the number of live children born and the number of surviving children. With this information, using the indirect method of own children we determined infantile mortality. Then, using the relational logit model and the model life table (west), we estimated the survival function for all ages.

**Results:**

Indigenous Chileans have seven years lower life expectancy at birth than the non-Indigenous population (76.2 vs. 83.2 years). The differential at age 60 is 6 years (20.3 vs. 26.4 years). We also found that Mapuche have an even greater disadvantage in survival than other ethnic groups. This is reflected in 2 years less life expectancy, both at birth and at 60 years.

**Discussion:**

Our results ratify the existence of marked ethnic-racial inequality in the extension of life in Chile and demonstrate a greater disadvantage in terms of survival of the Mapuche compared to other Indigenous and non-Indigenous groups. It is thus of great relevance to design policies that would decrease the existing inequalities in lifespan.

## 1. Introduction

Prolonging the number of years of life is one of the greatest achievements of humans. However, although the population has benefited from the increase in life expectancy in the last decades in almost all regions, including Latin America, important differences have been detected in ethnic-racial health and longevity (1–7). Studies in other societies, for example Australia, New Zealand, Canada, and the USA, have detected differences in life expectancy at birth that range from 6 to 15 years between Indigenous and non-Indigenous people (7–9) while results for Latin America suggest lower differences (1, 10, 11).

The demographic changes associated with the transition of mortality and fertility in Chile have contributed to a considerable increase in life expectancy at birth in the last decades, which is currently 81.2 years (12). Advances in medicine, improvement and widening of the coverage of health services, social and economic progress are among the factors described that have favored this increase in survival of the population (12, 13). Chile currently has a population of 2.2 million Indigenous people, and of the 10 Indigenous group recognized by law, the Mapuche^1^ are the largest group (80% of the country's total Indigenous population). About 80% of the Indigenous population lives in urban areas and practically 50% live in the Metropolitan region, which is the most populated in the country. In socioeconomic terms in Latin American countries -including Chile- the inequality is structural and that it particularly affects Indigenous peoples (14–16). Thus, Indigenous Chileans have higher levels of poverty and indigence compared to non-Indigenous people (17–19). For example, 17.1% of Indigenous people live in poverty compared to 13.9% of non-Indigenous people (19). Further Indigenous people have higher death rates, including infant mortality (1, 20–23), respiratory diseases (24), trauma (21, 22) and SARS-CoV-2 (25); higher prevalence of chronic illnesses such as diabetes (26), more mental health problems, including depression and dementia (27, 28), as well as greater inflammation of biomarkers, for example IL-8 (29).

Summarizing, a limited number of studies have explored some central elements related to the health and longevity of the Chilean population, focusing the study on ethnic differences. This may be explained in part by a lack of information, like not including ethnic origin in the vital statistics of the country (30), and by the lack of reliable and representative information on the Indigenous populations in the surveys of national representability. In fact, the lack of statistical visibility of Indigenous people can be understood as another expression of discrimination and structural racism (31, 32).

Thus, in Chile there is still little knowledge about ethnic differences in life expectancy. This study asks if there are ethnic differences in life expectancy at birth and at age 60 years in Chile, and if the Mapuche (largest ethnic group) have similar life expectancy to other Indigenous groups. Answering these questions would provide information that could be very useful in designing public health policy, as well as promoting the study of differences in longevity in the entire country and in different parts of it. We believe that the increase in life expectancy in Chile has not benefitted all its inhabitants equally, that is, they have not been distributed homogenously in the population. We expect to find greater life expectancy at birth in the non-Indigenous population.

## 2. Methods

The data are from the Population and Housing Census of 2017 (33), which is freely accessible. Chilean law currently recognizes the existence of ten Indigenous peoples; the Mapuche are the most numerous, followed by the Aymara and Rapa Nui (see Table 1). For this study we first divided the population into Indigenous and non-Indigenous individuals, according to what they declared in the census. Since Mapuche are almost 80% of the Indigenous people of the country, we separated them from the others, who were grouped as “other Indigenous peoples”.

**Table 1:** General characteristics of the Chilean population according to ethnic-racial self-identification.

**Ethnic group**	**Population**		**Sociodemographic indicators**		
	**N**°	**%**	**% 60**+ **years**	**% with low education (**<**8 years)**	**% living in rural area**
**Indigenous**	**2,185,792**	**12.44**	**13.5**	**27.67**	**19.4**
Mapuche	1,745,147	9.93	13.8	29.19	20.66
Aymara	156,754	0.89	10.8	19.77	12.50
Rapa Nui	9,399	0.05	13.1	20.54	7.93
Other Indigenous	274,492	1.56	13.0	23.55	17.14
**Non-Indigenous**	**14,890,284**	**84.73**	**16.7**	**22.56**	**11.13**
Missing ethnic identification	497,927	2.83	…	…	…
**Total**	**17,574,003**	**100.00**	**16.2**	**23.02**	**12.23**

Source: Own elaboration based on the 2017 census.

### 2.1. Analytic strategy

To generate life tables according to ethnic origin, we first estimated infant mortality by the method of Brass (27). This method allows converting the proportions of child deaths among all children born alive declared by women aged 15–49 years into estimations of the probability of dying before reaching certain ages (34, 35). The information required is the number of live births and the number of living children classified by 5-year age groups of the mothers, provided by the census. The Brass method has been widely used when information on age-specific mortality rates is not available and is quite robust to non-compliance with its assumptions (constant mortality and fertility and closed population) (36).

It must be noted that Chile does not currently incorporate ethnic origin in its vital statistics, which makes it impossible to obtain directly mortality rates by age according to ethnic origin. It is difficult to use other indirect mortality estimates such as the inter-census method, due to changes in the questions over time. In the previous valid census, of the year 2002, people were asked if they were members of an Indigenous group^2^, while in 2017, people were asked to self-identify their ethnicity^3^. This change makes it difficult to compare estimations over time, thus we decided to use only the 2017 census. It is important to note that the question on ethnic self-identification is answered by each person over 15 years old. For minors and those who do not have the cognitive abilities to answer by themselves, the adult person selected as informant is the one who reports the ethnicity.

Then we used a relational logit model with a model table (of the Princeton West family) to construct life tables for the Indigenous and non-Indigenous populations. Since we only had information for the indirect estimation of infant mortality and considering that population ageing is importantly due to decrease in mortality, especially in older age groups, in the process of constructing life tables using ethnic-racial self-identification we detected irregularities in older age groups (70 years and older).

**Figure 1:**
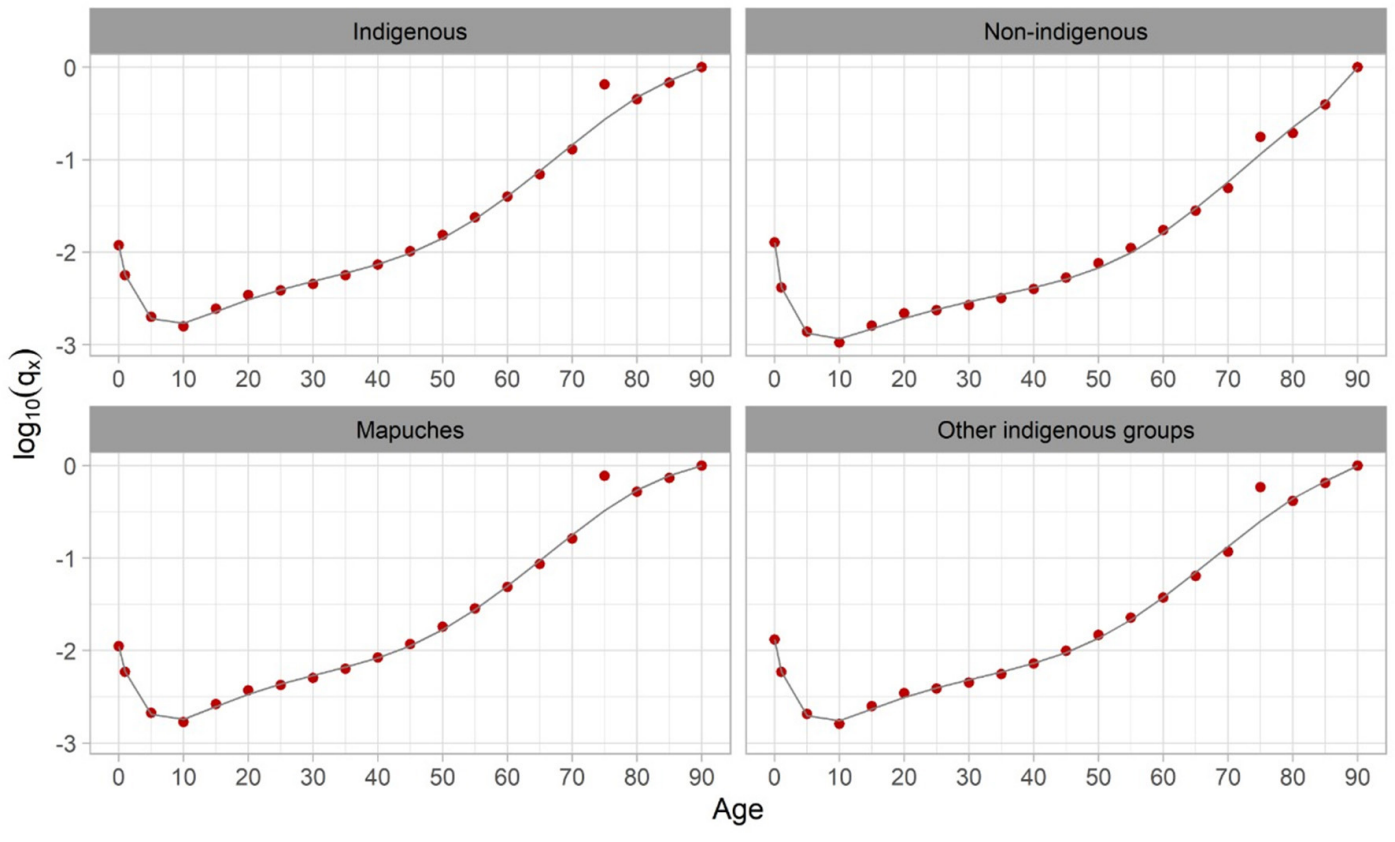
Logarithms of adjusted observed survival probabilities. Source: Own Elaboration.

Although the 2017 Census presents good indicators of age reporting (e.g., Whipple Index <105), it is likely that detected irregularities may be related to the existence of an age differential bias in ethnic self-identification. To correct the irregularities detected in older ages, we performed a smoothing process using the Heligman-Pollard formula as a tool for expanding an abridged life table proposed by Kostaki (37), and the method of Arriaga (38) to obtain the adjusted life tables. Figure 1 shows the observed adjusted probabilities of death using the procedures just described. It may be seen that the described irregularities were in the 75–80 age group.

Finally, since the census asked about the total number of children born alive and the total number of surviving children, not distinguishing by gender, our estimations refer to the life expectancy at birth for both sexes together.

## 3. Results

Table 1 provides the general characteristics of the Chilean population by self-identified ethnic-racial origin according to the 2017 census. The proportion of the population that self-identified as Indigenous was 12.4%; 84.7% did not so identify. Older adults comprised 13.5% of the Indigenous population; this percentage was 16.7% among the non-Indigenous people.

Table 1 also indicates that 27.7% of Indigenous people had 8 or fewer years of school, which is greater than that of non-Indigenous Chileans, and that 19% of the Indigenous population lives in rural areas. The estimations of the life tables by ethnic-racial origin show that the Indigenous population of Chile has a life expectancy at birth of 76.2 years, while for the non-Indigenous population it is 83.2 years (Table 2). These findings corroborate our working hypothesis, that life expectancy at birth is greater for non-Indigenous persons. Table 2 also shows that the life expectancy disadvantage for the Indigenous population is maintained during almost the entire life span. For example, at age 60 the non-Indigenous life expectancy was 6 years greater (26.4 years compared to 20.3 years for Indigenous people), and at 85 years there was still a difference of 2.5 years (6.6 and 4.1 years, respectively).

**Table 2:** Life expectancy by age and ethnicity in Chile.

**Age**	**Indigenous (a)**	**Non-Indigenous (b)**	**Mapuches (c)**	**Other indigenous group (d)**	D^*^(b–a)	D^*^(d–c)
0	76.2	83.2	74.6	76.7	7.0	2.1
1	76.1	83.3	74.5	76.8	7.1	2.3
5	72.6	79.6	70.9	73.2	7.0	2.3
10	67.7	74.7	66.1	68.3	7.0	2.3
15	62.8	69.8	61.2	63.5	7.0	2.3
20	58.0	64.9	56.3	58.6	6.9	2.3
25	53.1	60.0	51.5	53.8	6.9	2.3
30	48.3	55.2	46.7	49.0	6.8	2.3
35	43.6	50.3	42.0	44.2	6.8	2.3
40	38.8	45.5	37.2	39.5	6.7	2.2
45	34.1	40.7	32.5	34.7	6.6	2.2
50	29.4	35.9	27.8	30.0	6.5	2.2
55	24.8	31.1	23.3	25.4	6.3	2.1
60	20.3	26.4	18.9	20.9	6.1	2.0
65	16.0	21.8	14.7	16.6	5.7	1.9
70	12.1	17.3	11.0	12.7	5.2	1.7
75	8.7	13.2	7.8	9.2	4.5	1.5
80	6.0	9,6	5.3	6.4	3.6	1.2
85	4.1	6,6	3.6	4.4	2.5	0.9
90	3.9	4.5	3.3	4.0	0.7	0.7

D^*^, Difference.

Source: Own estimates using the 2017 census.

The results shown in Table 2 also demonstrate that Mapuche have a life expectancy at birth 2.1 years less than other Indigenous groups as a whole and 8.3 years less than non-Indigenous people. At 60 years, the difference with other Indigenous groups is 2 years, and 7.5 years less than non-Indigenous people.

It is important to note that the differences between Mapuche and the other Indigenous groups, and between Mapuche and non-Indigenous groups remain very similar during the whole life cycle. As the figure indicates, the difference only reduces at 80 years. Also, the other Indigenous groups have lower life span than non-Indigenous people. An Indigenous non-Mapuche has a life expectancy at birth 6.5 years less than non-Indigenous people, and 5.5 years less at age 60.

## 4. Discussion

Life expectancy at birth has been used frequently as an indicator of inequality; this study was designed to contribute to clarify the ethnic differences in life span in Chile. Using the 2017 Chilean census and indirect demographic methods, we show that Indigenous peoples have seven years lower life expectancy at birth than the non-Indigenous population. Indirect methods have been used widely to estimate mortality in data-deficient societies and populations and to estimate ethnic-racial differences in mortality (20, 36, 39–41).

Our results demonstrate that Indigenous people in Chile, one of the most developed countries in the region, on average live seven fewer years than non-Indigenous people. Most of the evidence that shows shorter life spans for Indigenous peoples come from industrialized countries. For example, Dankovchik et al. (42) found that in the USA, specifically in the Pacific Northwest and Alaska, American Indians and Alaska Natives have a life expectancy at birth 6.9 years less than non-Hispanic whites. Omisakin et al. (43) found that in the Four Corners States (Arizona, Colorado, New Mexico, and Utah) the difference in life expectancy between Native Americans and whites reduced in 2001–2012, however, it then increased, reaching 4.92 years for men and 2.06 years for women in 2015. Tjepkema et al. (9) showed that life expectancy at age 1 year in Canada for men of the First Nations was 72.5 years, 76.9 years for the Métis and 70 years for the Inuit, while it was 81.4 years for the non-Indigenous people. Similar differences were found for women in the same ethnic groups (77.7, 82.3, 76.1 and 87.3 years, respectively).

An important number of studies have documented large differences in life expectancy at birth between Indigenous and non-Indigenous people in Australia and New Zealand, from 13 to 20 years, depending on the methodology used (4, 8, 44, 45). For example, Hill et al. (4) used adjusted life tables and found a difference of 13 years. A recent study of Zhao et al. (7) in the Northern Territory of Australia found a reduction in the difference in life expectancy at birth of 26% for men and 21% for women in the 1999–2018 period, although there continues to be a substantial difference between the groups. Anderson et al. (1) compared countries with low, medium, and high average income, finding differences that ranged from 0.1 years in Nepal (Sherpa, Rai Magar, Tamanga) to 21.5 years in Cameroon (Baka people). All these results show that life expectancy is consistently lower for Indigenous than for non-Indigenous people.

In Latin America, Robles (11) used the indirect method of orphans to show that in Guatemala Indigenous women aged 20 years had 3.2 years lower life expectancy than non-Indigenous people (46.8 and 50.0 years, respectively) at the end of the 1980s. Similarly, Chackiel (36) found ethnic differences in life expectancy at birth in Panama, translating into a 11-year disadvantage for indigenous women (63.6y and 75.1, respectively) and an 8-year disadvantage for indigenous men (61.1y and 69.6y, respectively). Further, Chiavegatto et al. (46) found that white women in Brazil have a life expectancy at birth 2.5 years greater than Blacks (78.8 and 76.3, respectively), while the difference among men is 1 year (71.1 vs. 70.0 years). Urrego (10) used the 2005 census of Columbia to estimate a difference in life expectancy at birth for Indigenous people of 5 years for men and 8.4 years for women. Finally, Anderson et al. (1) found a difference in life expectancy at birth of 7.6 years between Indigenous and non-Indigenous people.

There are several factors that may explain these differences. A structural mechanism may be involved; in different societies (including Chile) low socioeconomic status is associated with worse health results and greater mortality (47, 48). Table 1 indicates that Chilean Indigenous people have important socioeconomic disadvantages; for example, while the Chilean population has an average of 12.8 years of schooling (49), 27.7% of indigenous people have <8 years of schooling, which may result in poorer life conditions and greater exposition to diseases that affect their chances of survival. Another important point is that the vast majority of the Indigenous population of Chile lives in urban areas. Between the middle of the 20th century and the end of the 1980s there was an important flow of Indigenous people to the urban centers of the country, mainly the Metropolitan Region; currently only 19.4% of Indigenous Chileans live in rural areas (see Table 1). It is known that moving from a rural to an urban area may produce changes in lifestyles of Indigenous people (3), along with greater exposition to unfavorable surroundings (precarious, marginal) which finally affect health and chances of survival.

Psychosocial and lifestyle factors (such as alcoholism, smoking and exercise) and the access to and use of health services also play an important role in explaining ethnic differences in longevity (Blakely et al. (2). In addition to individual factors, the health of the indigenous population is certainly affected by contextual or environmental factors. The territory determines opportunities and socioeconomic conditions that affect the realization of political, economic, and social rights of the indigenous population, which intersect, deepening the deprivation of rights (50) and, certainly, affecting the health of the indigenous population. Thus, the reduction of indigenous territories and the forced displacement and internal migration of indigenous people to intermediate cities and regional capitals could certainly be part of the health disadvantages of indigenous people.

The marked longevity differences between Mapuche and other Indigenous groups show that Mapuche have lower life expectancy. This has at least three possible explanations, independent or combined. We recognize that the history of Original Peoples in Chile, as in other societies, was marked by processes of oppression, marginalization, and discrimination, which no doubt may affect health and thus reduce the chance of a long life for Indigenous people. It is probable that this has affected mainly the Mapuche population in Chile, which has been the victim of an inadequate recognition and implementation—without cultural relevance of their civil, political, social and cultural rights by the Chilean State. Second, the process of re-ethnicization that has been occurring in the country in recent years may be affecting the composition of the minor Indigenous groups; “contemporary Indigenous” individuals who formerly did not recognize themselves as such may have different characteristics than “traditional Indigenous” people, for example more years of education. At the same time, in contexts of discrimination and structural racism, such as that suffered by Indigenous people in urban contexts, it is possible that Indigenous people in less favorable situations do not self-identify as such, which may even vary among the different ethnic groups in the country, also affecting the composition of ethnic minorities. This may influence the results and increase the survival of these Indigenous groups. Third, part of the difference may be associated with the data source and methodology used in this study, due to which it is important to explore other sources and methodologies that would allow re-estimating life expectancy according to ethnic origin in the country. These and other possible explanations certainly need to be evaluated in future work. However, our study provides the first evidence of ethnic-racial differences in life expectancy in Chile with data for the entire population of the country.

In Latin America, the inclusion and measurement of ethnic categories within official statistics stems from socio-historical processes of conquest, colonization, and expansion of republican states in the region, the result of negotiations and dynamic tensions in power relations between social forces and public institutions (51, 52) and has therefore been accompanied by an important debate about the social, economic, and political implications of the time. The measurement and provision of data on ethnic groups has not been easy in Latin American societies, where ethnic diversity has historically been denied as one of its main characteristics (52). In this sense, the construction of the question for the inclusion of the ethnic approach has undergone significant variations over time in accordance with advances in the recognition of the pluricultural nature of societies. Although we cannot speak of a definitive standard measure, it can be said that until now it has been the self-identification questions that have been used most frequently to identify Indigenous groups in the region, including Chile. The self-identification question is based on the fact that awareness of their Indigenous or tribal identity is the fundamental criterion for identification (31, 53), and it is therefore considered that it is the peoples and individuals who consider themselves Indigenous who must define themselves as such, making it essential to recognize the right to self-identification as part of the right to self-determination (54). Therefore, in Latin America—including the Chilean case—the identification of indigenous peoples is based on the criterion of self-identification, which emerges as a right to self-determination and not on genetic components.

This study has several limitations, including the data source used. It is probable that the ethnic differences in life span in Chile are either underestimated or overestimated. Since there is no source of administrative data (for example, vital statistics) that would allow a direct comparison of Indigenous to non-Indigenous people, it is hard to know exactly the size of the gap in survival and longevity. In the future, data recording in Chile should include the ethnic origin variable in the vital statistics, as Sandoval and Alvear (30) suggested. This would help researchers to understand the “why?” and “how and/or what?” of the deaths of people by ethnic origin over time, facilitating the monitoring of ethnic differences in health and mortality.

A second limitation, related to the above, is that the life tables of the Indigenous—including the Mapuche—and non-Indigenous people were elaborated using only one parameter, in this case infant mortality. It is probable that a single parameter would not fit well to the calendar of mortality by age of a population. However, we feel that our results are a first advance towards the study of ethnic differences in longevity in Chile. Using indirect demographic methods, designed specifically for contexts with lacking information, we found a difference of 7 years in the life expectancy of birth between Indigenous and non-Indigenous people.

The results obtained in this study are of ethnic differences in life expectancy for both sexes together. Unfortunately, the census did not include questions about the sex of children born alive and survivors, thus it was impossible to make estimations separately for males and females. Future studies must find a way to examine gender and ethnic origin differences in life expectancy.

This study ratifies the existence of marked ethnic-racial differences in life span in Chile. The country has made significant advances in recognizing the multi-cultural character of its population, for example the program for recovery and revitalizing Indigenous languages begun in 2006 (55), the program of bilingual intercultural education since 1996 (56) and the inclusion of intercultural aspects in health services (57). However, the results of this study demonstrate the need to generate and execute initiatives that will help to diminish the ethnic-racial inequalities in survival. This is in line with what was established in the Objetivos Sanitarios de la Década 2020–2030 (58) (health objectives for the 2020–2030 decade) of the Chilean government, which propose to “improve the health and well-being of the population” and “decrease the inequalities” in health.

Finally, it is probable that the ethnic-racial differences in life expectancy in Chile are even greater, as has been detected in societies in which relatively good-quality data on ethnic-racial origin and mortality have been recorded for many years. Thus, it is relevant for future studies to continue exploring variations in longevity according to ethnicity, to determine if these are maintained, increase, or decrease, and to advance in the study of the factors associated with these differences.

## Data availability statement

Publicly available datasets were analyzed in this study. This data can be found here: https://www.ine.gob.cl/estadisticas/sociales/censos-de-poblacion-y-vivienda.

## Ethics statement

Ethical review and approval was not required for the study on human participants in accordance with the local legislation and institutional requirements. Written informed consent for participation was not required for this study in accordance with the national legislation and the institutional requirements.

## Author contributions

MS and MA: study design, conceptual interpretation, data analysis, interpretation, and manuscript drafting and reviewing. CA: drafting and manuscript reviewing. All authors contributed to the article and approved the submitted version.

## Footnotes

^1^Plural term meaning people of the land.

^2^Included the question: Do you belong to one of the following Original or Indigenous peoples?

^3^Included two questions: 1) Do you consider that you belong to an Original or Indigenous people? If your answer is yes, 2) To which one?
